# Effects of Calorie Restricted Diet on Oxidative/Antioxidative Status Biomarkers and Serum Fibroblast Growth Factor 21 Levels in Nonalcoholic Fatty Liver Disease Patients: A Randomized, Controlled Clinical Trial

**DOI:** 10.3390/nu14122509

**Published:** 2022-06-16

**Authors:** Somayyeh Asghari, Mahsa Rezaei, Maryam Rafraf, Mahdiyeh Taghizadeh, Mohammad Asghari-Jafarabadi, Maryam Ebadi

**Affiliations:** 1 Department of Clinical Nutrition, Faculty of Nutritional Sciences and Dietetics, Tehran University of Medical Sciences, Tehran 141556117, Iran; sasghari@sina.tums.ac.ir (S.A.); mahsarezaei1372@gmail.com (M.R.); mahdiyehtaghizadeh9969@gmail.com (M.T.); 2 Nutrition Research Center, Department of Community Nutrition, Faculty of Nutrition and Food Science, Tabriz University of Medical Sciences, Tabriz 5166614711, Iran; rafrafm@tbzmed.ac.ir; 3 Department of Statistics and Epidemiology, Faculty of Health, Tabriz University of Medical Sciences, Tabriz 5166614711, Iran; m.asghari862@gmail.com; 4 Cabrini Research, Cabrini Health, 154 Wattletree Rd, Malvern, VIC 3144, Australia; 5 Division of Gastroenterology & Liver Unit, University of Alberta, Edmonton, AB T6G 2X8, Canada

**Keywords:** NAFLD, calorie restriction, clinical trial, oxidative stress, fibroblast growth factor 21

## Abstract

Oxidative stress plays a fundamental role in the development and progression of nonalcoholic fatty liver disease (NAFLD). This study aimed to investigate the effects of a calorie-restricted (CR) diet on oxidative/anti-oxidative status in patients with NAFLD and the potential mediating role of fibroblast growth factor 21 (FGF-21) in this regard. This randomized, controlled clinical trial was carried out on sixty patients with NAFLD aged 20 to 60 years with body mass index (BMI) ranging from 25 to 35 kg/m^2^. Participants were randomly assigned to either the CR diet group (received a prescribed low-calorie diet for twelve weeks, *n* = 30) or the control group (*n* = 30). Fasting blood samples, anthropometric measurements, dietary intake, and physical activity data were collected for all participants at baseline and at the end of the trial. Significant reductions in weight, BMI, waist circumference, and serum alanine aminotransferase (ALT) and aspartate aminotransferase (AST) were observed in the CR diet group compared to the control group (all *p* < 0.05). Liver steatosis grade, serum levels of malondialdehyde (MDA), total antioxidant capacity (TAC), and FGF-21, as well as erythrocyte superoxide dismutase (SOD) and glutathione peroxidase (GSH-Px) activities did not show significant changes in the CR group when compared to the controls at the end of the study (*p* > 0.05). CR diet with moderate weight loss has some favorable effects on NAFLD but was not able to modify oxidative/anti-oxidative status in these patients. Future studies are warranted to target the effects of long-term interventions with a greater weight loss in this patient population.

## 1. Introduction

Non-alcoholic fatty liver disease (NAFLD) has become the leading cause of chronic liver disease worldwide [1]. The spectrum of NAFLD ranges from simple steatosis to nonalcoholic steatohepatitis, which may progress to cirrhosis and hepatocellular carcinoma [1,2]. The current prevalence of NAFLD in the general population is around 25% [1], given the rising prevalence of obesity, type 2 diabetes mellitus, and metabolic syndrome as well as a robust relationship between NAFLD and metabolic disorders. The prevalence of NAFLD is estimated to be higher in the near future resulting in a huge economic and clinical burden on patients and the health care systems [1,2]. Insulin resistance is the main pathological factor responsible for the initiation and progression of NAFLD [3]. In an insulin resistance state, elevated free fatty acid efflux from adipose tissue into circulation causes lipotoxicity in the liver. This can result in increased mitochondrial beta-oxidation, reactive oxygen species (ROS) overproduction, and massive oxidative stress [3,4,5]. Oxidative stress is involved in NAFLD advancement from simple steatosis to steatohepatitis and hepatic failure, as well as increasing the risk of several chronic diseases such as type 2 diabetes and cardiovascular disease [4,5,6]. Previous research has shown increased levels of circulating lipid peroxidation products and decreased antioxidant levels in patients with NAFLD [7,8].

Currently, there is no specific pharmacotherapy for NAFLD [9]. Diet and exercise as lifestyle modifications have been considered the first-line therapies for NAFLD management [10,11]. According to recent reports, a calorie-restricted (CR) diet while maintaining sufficient nutrition, ameliorates oxidative stress in overweight and obese patients [6]. However, the exact molecular mechanisms underlying the impacts of a restricted-calorie diet on oxidative stress alleviation are not completely elucidated [12]. Fibroblast growth factor 21 (FGF-21), a new endocrine hormone with the regulatory effects on energy homeostasis and metabolism of lipid and glucose, has been known to inhibit the inflammation and cell damage caused by oxidative stress in obesity and insulin-resistant states including NAFLD [13]. There is some evidence suggesting a role of the FGF-21 axis in the beneficial effects of the CR diet [14]. While earlier rodent studies found rapid increment in circulating FGF-21 and hepatic FGF-21 mRNA levels in response to fasting [15], human data demonstrated higher levels of FGF-21 in obesity-related conditions like NAFLD compared to the healthy controls [14] suggesting the presence of FGF-21 resistance in NAFLD [14,16]. In line with this statement, the CR diet lowered serum FGF-21 levels and increased the expression of FGF-21 receptors in hyperphagic rats indicating a reduction in FGF-21 resistance [17]. Although associations between CR and FGF-21 have been investigated, data regarding the impact of the CR diet on circulating FGF-21 levels and the biomarkers of oxidative stress in NAFLD patients is lacking. Thus, the aim of the present study was to investigate the impacts of the CR diet on the status of oxidative stress in NAFLD patients and the possible involvement of FGF-21 in this respect.

## 2. Materials and Methods

The ethics committee of the Tabriz University of Medical Sciences (TBZMED.REC.1394.823) approved this study which was performed in compliance with the principles of the Declaration of Helsinki. The trial was registered at the Iranian Registry of Clinical Trials (registration no. IRCT201511233664N16). This study follows the Consolidated Standards of Reporting Trials (CONSORT) reporting guideline (Table S1). Prior to the study enrollment, written informed consent was obtained from all participants.

### 2.1. Study Population

Sixty adult NAFLD patients aged 20 to 60 years old and body mass index (BMI) values between 25 and 35 kg/m^2^ were included in this study. All patients were assessed in the Golgasht outpatient clinic at the University of Tabriz (Iran) from January to June 2016. NAFLD diagnosis was performed using liver ultrasonography at baseline and at the end of the study by the same radiologist using the SonoAce X4 ultrasound machine (Medison Inc., Seongnam, Korea 2009). Hepatic steatosis grade was determined according to the macrovesicular steatosis and divided into four categories of grade 0, grade 1, grade 2, and grade 3 [18].

Patients were not included in the study in cases of pregnancy; breastfeeding; postmenopausal status; smoking; being a professional athlete; alcohol consumption; following a restricted-calorie diet up to three months prior to the study; intake of medications including oral antidiabetics, hepatotoxic or lipid-lowering drugs, corticosteroids, hormonal medications (e.g., oral contraceptives), anticoagulants, antidepressants, or psychotropic drugs; consumption of any type of supplements three months before the study and/or during the study period; history or current diagnosis of liver disease (viral, inherited disorders, etc.); thyroid dysfunction; cardiovascular disease; cancer; pulmonary disease; diabetes; renal disease; gastrointestinal disorders; and autoimmune diseases.

### 2.2. Study Design

This study was a randomized, controlled clinical trial in which 60 patients were randomly allocated into two intervention groups using a block randomization method stratified by age, sex, and BMI. A random sequence was generated using the random allocation software (RAS). The CR group (*n* = 30) underwent a calorie restriction treatment and the control group (*n* = 30) received healthy eating and weight control advice for 12 weeks. Participants in the CR group were targeted to lose a maximum of 10% of their baseline body weight through a healthy calorie-restricted diet. The Harris-Benedict equation was used for the estimation of patients’ energy requirements individually. To restrict calories, 500 to 1000 kcal/day (depending on body weight) was deducted from the estimated energy requirements. The prescribed diets contained 17% protein, 30% fat, and 53% carbohydrate which were planned individually based on the optimal number of servings of each food group from the Food Guide Pyramid as well as subjects’ food preferences and allergies, or intolerances. A dietitian fully informed participants of both groups about the healthy eating fundamentals according to the Food Guide Pyramid and the recommendations of the Obesity Education Initiative Expert Panel. We conducted individual interviews during follow-up visits every two weeks to assess adherence to the prescribed diet plan, discuss and resolve any barriers or concerns related to the diet and encourage compliance. Missing more than two appointments or failing to lose more than 2% of baseline weight was considered noncompliance.

### 2.3. Study Measurements

Anthropometric measurements were done for all patients at the beginning and the end of the study. Body weight was recorded in light clothing without shoes using a scale (Seca, Hamburg, Germany), to the nearest 0.5 kg. A barefoot measured height was determined using a mounting tape with a precision of 0.1 cm. Body mass index (BMI) was calculated as weight (kg) divided by the squared height (m^2^). Waist circumference (WC) was measured using an unstretched measuring tape placed at the closest line below the ribs. Hip circumference was measured at the largest level under the waist.

Food record questionnaires for three days (two weekdays and one weekend day) were used for dietary assessment in the week just before the study and week 12. The analysis of dietary intakes was conducted using a modified Nutritionist 4 software (First Databank, Hearst Corp., San Bruno, CA, USA). The International Physical Activity Questionnaire was used for assessing physical activity levels at the beginning and the end. The liver ultrasonography was performed by the same blinded examiner in a single center before and after the intervention.

Twelve-hour fasting blood samples (10cc) were collected at the beginning and end of the intervention period. Samples were centrifuged at 3500 rpm for 10 min to separate the serum. EDTA collection tubes were also used to stabilize the whole blood samples. Both whole blood and serum samples were promptly kept at −70 °C until carrying out the analysis. All biochemical tests were performed at the Drug Applied Research Center of the Tabriz University of Medical Sciences, Tabriz, Iran. Serum liver enzymes including aspartate aminotransferase (AST) and alanine aminotransferase (ALT) were measured by an enzymatic method (Pars Azmoon Inc. kit, Tehran, Iran) using an autoanalyzer set (Alcyon 300, USA). Thiobarbituric acid reactive substances (TBARS) assay, a method explained by Bilici et al., was used to determine serum malondialdehyde (MDA) concentration [19]. Total antioxidant capacity (TAC) was determined by the Randox kit (Randox Laboratories, Ltd., Crumlin, UK) using the spectrophotometry method. Erythrocyte glutathione peroxidase (GSH-Px) and superoxide dismutase (SOD) activities were measured using Ransel and Ransod kits, respectively (Randox Laboratories, Ltd., Crumlin, UK). Results were stated as units of GSH-Px and SOD activity per milligram of hemoglobin. The commercial cyanmethemoglobin method (Pars Azmoon Inc. kit, Tehran, Iran) was used for hemoglobin measurement in the hemolysates. Fasting blood sugar (FBS) levels were measured by the glucose oxidase enzymatic method (Pars Azmoon Inc. kit, Tehran, Iran). Serum total cholesterol (TC), triglycerides (TG), and high-density lipoprotein cholesterol (HDL-C) were measured using an enzymatic method (Pars Azmoon Inc. kit, Tehran, Iran). Low-density lipoprotein cholesterol (LDL-C) concentration was calculated by Friedewald’s formula (LDL-C = TC-HDLC-TG/5). Circulatory FGF-21 was assessed by ELISA kit (Human FGF-21 ELISA kit, Crystal Day Bio-Tec) with a detection limit of 5 pg/mL.

### 2.4. Statistical Analyses

SPSS software version 17 (SPSS Inc., Chicago, IL, USA) was used to perform all statistical analyses. The Kolmogorov-Smirnov test was used to assess the normality of the distribution of variables. Accordingly, non-normal variables were analyzed following a log transformation. Independent samples *t*-test and chi-squared test were performed in order to compare baseline characteristics where appropriate. Within-group comparisons were conducted using a paired samples *t*-test. Analysis of covariance (ANCOVA) was applied to determine the impact of the intervention when adjusted for baseline values and physical activity level changes. Categorical variables were reported as frequency (percentage), numeric normal variables as mean (standard deviation [SD]), and numeric non-normal variables as geometric mean (minimum, maximum). In all analyses, *p* < 0.05 was considered statistically significant.

## 3. Results

**Figure 1:**
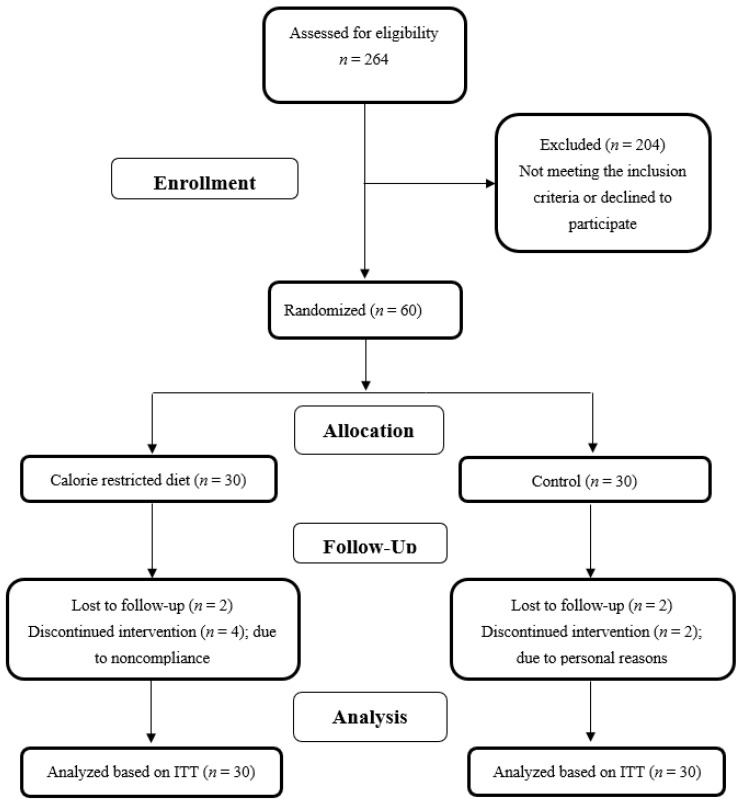
Study flow diagram.

Among sixty patients included in the study, twenty-four in the CR group and twenty-six controls completed the 12-week intervention. Figure 1 summarizes the study flow diagram explaining the reasons for the participant dropouts in each group.

### 3.1. General Characteristics of the Participants

The baseline characteristics of the study participants are summarized in Table 1. The distribution of age and sex were homogeneous between the two groups at baseline (*p* > 0.05). At baseline, no significant difference in weight, BMI, WC, grade of liver steatosis, and physical activity level was observed between the CR and control groups (Table 1). Following 12 weeks intervention, weight (MD = −4.06; 95% CI [−4.50, −3.72]; *p* < 0.01), BMI (MD = −1.38; 95% CI [−1.50, −1.23]; *p* < 0.01), and WC (MD = −4.14; 95% CI [−4.50, −3.80]; *p* < 0.01) reduced significantly in the CR group compared to the control group.

Physical activity levels did not change significantly throughout the study neither within, nor between the two groups (*p* > 0.05).

### 3.2. Dietary Intakes

Energy, macronutrients, and antioxidant micronutrient intake did not differ between the two groups at baseline. There was a 361.40 ± 16.83 (mean ± SD) kcal decrease (−16.6%) in the daily energy intake of participants in the CR group during 12 weeks of intervention (*p* < 0.01). Dietary carbohydrate, fat, and protein intakes decreased by 20%, 12%, and 10% during the intervention in the CR group, respectively (*p* < 0.01). In contrast, there were no major changes in energy (−0.67%) or macronutrient intakes (−1.06% for carbohydrates, −2.52 for protein, and −1.14% for fat) in the controls throughout the study (*p* > 0.05). The changes in energy and macronutrient intakes were significantly different between the two groups at the end of the study (*p* < 0.01). Patients’ dietary intakes were reported in detail in Asghari et al. (2018) [20]. No significant difference in dietary antioxidant intakes was noticed between the two groups throughout the study (Table 2).

Participants’ compliance to the prescribed diet plan in the CR group was relatively moderate, as we planned an energy deficit of 540 kcal on average, but there was an approximately 360 kcal decrease in daily energy intake of participants. Thus, the compliance rate is calculated to be 67%. However, only four subjects could not lose more than 2% of their baseline weight.

### 3.3. Biochemical Parameters

Table 3 presents the Biochemical parameters and liver steatosis grade of patients throughout the study.

There were no significant differences in biochemical values between the study groups at the baseline. After the intervention, serum levels of liver enzymes including ALT and AST reduced considerably in the CR group in comparison to the control group (*p* < 0.05).

Serum levels of FGF-21, MDA, and TAC, as well as erythrocyte SOD and GSH-Px activities, did not show significant changes in the CR group when compared to the changes in controls at the end of the study (*p* > 0.05).

While FBS, TG, LDL-C, and HDL-C levels did not change significantly in the CR group when compared to the control group (*p* > 0.05), a significant difference was observed in TC levels (*p* = 0.03).

At the end of the study, no significant changes were observed in patients’ liver steatosis grades either within or between the two groups (*p* > 0.05), although two patients in the CR diet group had a decreased steatosis grade and one patient in the control group had an increased steatosis grade.

## 4. Discussion

To our knowledge, this trial is the first to investigate the effects of a moderately calorie-restricted diet on FGF-21 levels in overweight or obese NAFLD patients. In the current trial, the CR diet was associated with a significant reduction in weight and other anthropometric indices when compared to the controls. Reduction in anthropometric parameters by calorie-restricted diets has been reported repeatedly in NAFLD patients [21,22,23]. An average weight loss of 5% in this study did not provide any significant improvement in hepatic steatosis grade. Thomas et al. reported a hepatic fat reduction in NAFLD patients following an average weight loss of 4% through a 500-kcal restricted diet, although this was not statistically significant [24]. The impact of weight loss on lowering the hepatic fat content in patients with NAFLD was demonstrated in other studies [25,26]. Tiikkainen et al. observed a significant decrease in hepatic fat content by an average of 8% weight loss in women with both high and low hepatic fat accumulation [27].

It has been suggested that losing 5% of initial body weight through lifestyle modification is needed for the reduction of hepatic steatosis. While losing 7–10% of initial body weight has been shown to be associated with a significant improvement in the NAFLD activity score [28]. It seems that the amounts of weight loss or the duration of CR diet intervention in our study were not adequate to reduce hepatic steatosis grade. However, the AST, ALT, and TC levels significantly improved in the CR group. This is in accordance with previous studies including meta-analyses showing the favorable effects of weight reduction and lifestyle interventions on NAFLD features [29,30,31].

Higher levels of oxidative stress biomarkers and diminished levels of both enzymatic and non-enzymatic antioxidants and their activities were found in patients with NAFLD [7,32]. Despite the strong role of oxidative stress in NAFLD pathogenesis and progression to NASH, there is limited evidence regarding the effect of CR diet and weight loss on markers of oxidative stress in NAFLD patients [3,5,21,33]. We measured the circulating levels of MDA and TAC, as well as erythrocyte GSH-Px and SOD activities to assess the impact of the CR diet on oxidative stress status in NAFLD patients. MDA is a well-known biomarker of oxidative stress indicating the peroxidation of polyunsaturated fatty acids by ROS [34]. Moreover, TAC represents the antioxidant capacity of body fluids which has been used as an oxidative stress biomarker in several pathological conditions [35,36]. SOD and GSH-Px are the most powerful antioxidant enzymes in the cell, which act as the first-line defense against ROS in biological systems [37]. Following twelve weeks of the CR diet and a moderate weight loss in this study, the levels of TAC and MDA, as well as erythrocyte SOD and GSH-Px, remained unchanged. While Ghaemi et al. found increased MDA levels in spite of weight reduction in patients with NASH [21], weight loss through the CR diet significantly decreased MDA levels and lipid peroxidation in an experimental model of NAFLD [33]. Few researchers have addressed the effect of weight loss on oxidative stress biomarkers in patients with NAFLD; however, it has been widely investigated in other populations [38,39,40,41]. Ramezani et al. reported a significant reduction in plasma MDA levels in obese women who lost their weight through CR diets with a daily deficit of 500–1000 kcal for twelve weeks [38]. Abd El-Kader et al. found that weight reduction induced by aerobic exercise and diet regimen resulted in a significant decrease in MDA and increase in SOD, GSH-Px, and GSH in obese patients with type-2 diabetes [39]. A significant decrease in plasma protein carbonyl, and a substantial increase in plasma GSH-Px activity with no changes in the activity of plasma SOD were observed in overweight volunteers [42]. Buchowski et al. also found decreased plasma F2-isoprostane concentrations, a biomarker of oxidative stress, during twenty-eight days of CR diet in premenopausal women with obesity [43]. In contrast to the previous study, a randomized controlled trial in patients with metabolic syndrome did not find any changes in urinary or plasma F2-isoprostane with a 4 kg weight loss over a twelve-week program [44].

FGF-21, a member of the growth factor family, is an endocrine hormone that increases energy expenditure to regulate energy homeostasis [45,46]. It is synthesized by several organs including the liver, pancreas, brown and white adipose tissue, and to a lesser degree by muscle. It has also multiple target tissues [46]. The liver is the main source of FGF-21 expression and also the major site of its actions [47]. In the liver, FGF-21 somehow adapts to the metabolic response to fasting or starvation states: gluconeogenesis, ketogenesis, and fatty acid oxidation [47]. Recently, studies have suggested FGF-21 as a novel regulator of oxidative stress. Increased levels of oxidative stress biomarkers are associated with FGF-21 upregulation which might be part of the potential defense strategies to avoid cell injury induced by oxidative stress [13]. On the other hand, in a previous study on patients with NAFLD, no changes in FGF-21 levels were observed depending on the degree of steatosis and oxidative damage markers [48].

The effects of calorie restrictions on FGF-21 levels are rather controversial. A comprehensive review by Crujeiras et al. concluded that a significant reduction in FGF-21 levels in CR diet is irrespective of the magnitude of weight loss in patients with obesity [49]. Elevated circulating levels of FGF-21 in insulin resistance states like obesity, type 2 diabetes, and NAFLD suggests an impairment and resistance to the actions of FGF-21 [46,50]. However, pharmacologic doses of FGF-21 were able to induce beneficial effects on liver function despite the presence of resistance to its actions in animal models [46]. To our knowledge, there is no previous data available considering the potential impacts of the CR diet on serum FGF-21 levels in NAFLD patients. We measured the levels of serum FGF-21 to examine the possible role of this hepatokine in mediating the impacts of the CR diet on oxidative stress status in NAFLD. However, no significant changes in serum FGF-21 levels were found after the CR diet intervention.

Various factors may contribute to non-significant results in this study. Firstly, the levels of oxidative stress biomarkers and antioxidant enzymes were within normal ranges at baseline. The severity of existing metabolic abnormalities before treatment seems to be of importance for measurable metabolic benefits of CR diet intervention. Accordingly, evaluating patients with levels of oxidative/antioxidative biomarkers outside the normal ranges would be necessary for these studies. Non-significant alterations in these biomarkers might also be partly associated with undetectable changes in serum FGF-21 levels. Secondly, the mean weight loss observed in previous studies with positive oxidative stress results was higher than the weight loss induced in this study. It is also worthwhile to note that the daily dietary consumptions of micronutrient antioxidants were not significantly different between the two groups throughout the study. Accordingly, dietary antioxidants are not expected to have confounded the results of this study. Overall, the discrepancies between the aforementioned studies and this study could be partially explained by various methodology (measuring different stress oxidative biomarkers), designs, and duration, as well as metabolic and clinical characteristics of the study participants.

We acknowledge several limitations of this study as the duration of intervention may not be long enough to observe alterations in the circulating levels of FGF-21 and oxidative stress biomarkers. Future studies are warranted to evaluate the effects of long-term (over a 3-month period) CR diets on FGF-21 levels. Compliance with the prescribed diet was relatively low to moderate in this study which did not allow patients to achieve a greater weight loss in order to determine its potential metabolic effects. In addition, using the Harris-Benedict equation for energy requirements instead of indirect calorimetry may overestimate the energy needs and result in higher energy intakes which could prevent greater weight loss.

## 5. Conclusions

CR diet along with moderate weight loss exerts some beneficial effects on liver function in patients with NAFLD; however, it was not able to modify serum levels of FGF-21 as well as oxidative/anti-oxidative status in these patients. Further studies are required to address the effects of long-term interventions with a greater weight loss in this patient population.

## Figures and Tables

**Table 1:** Baseline characteristics of the study patients.

Variables	Control (*n* = 30)	Calorie-Restricted (CR) (*n* = 30)	*p*-Value
Age (year)	39.27 (5.51)	40.08 (7.08)	0.90 ^†^
Sex			0.83 ^‡^
Male	19 (63%)	20 (67%)	
Female	11 (37%)	10 (33%)	
Weight (kg)	86.61 (10.70)	89.62 (14.20)	0.10 ^†^
BMI (kg/m^2^)	30.41 (3.39)	31.32 (3.31)	0.21 ^†^
Waist circumference (cm)	101.88 (8.18)	103.93 (10.30)	0.12 ^†^
Energy intake (kcal)	2082.6 (246.2)	2182.2 (268.7)	0.43 ^†^
Carbohydrate intake (g)	321.4 (47.8)	339.5 (50.9)	0.42 ^†^
Protein intake (g)	83.2 (5.7)	84.6 (6.2)	0.55 ^†^
Total fat intake (g)	52.8 (3.9)	53.9 (4.6)	0.51 ^†^
Physical activity level			0.63 ^§^
Light	15 (50%)	15 (50%)	
Moderate	15 (50%)	13 (43%)	
Vigorous	0 (0%)	2 (7%)	

Data are expressed as mean (standard deviation) or *n* (%). BMI: body mass index. ^†^ Independent *t*-test. ^‡^ Pearson chi-squared test. ^§^ Fisher’s exact test.

**Table 2:** Daily dietary intakes of known antioxidants of the study patients at baseline and after the 12-wk intervention.

Variables		Control (*n* = 30)	Calorie-Restricted (CR) (*n* = 30)	*p*-Value
Vitamin E (mg/day)	Before	10.53 (4.32)	9.87 (4.45)	0.46 ^†^
	After	8.96 (6.56)	7.59 (3.56)	0.12 ^‡^
	*p*-value ^§^	0.28	0.09	
Vitamin C (mg/day)	Before	86.42 (38.69)	83.43 (39.67)	0.83 ^†^
	After	90.33 (42.76)	93.35 (46.45)	0.26 ^‡^
	*p*-value ^§^	0.71	0.12	
β-Carotene (µg/day)	Before	566.17 (231.45)	576.65 (252.25)	0.88 ^†^
	After	623.14 (244.20)	651.12 (263.14)	0.61 ^‡^
	*p*-value ^§^	0.36	0.13	
Zinc (mg/day)	Before	8.09 (3.03)	7.95 (3.24)	0.61 ^†^
	After	8.28 (3.13)	7.37 (3.38)	0.14 ^‡^
	*p*-value ^§^	0.81	0.26	
Selenium (µg/day)	Before	67.33 (28.50)	68.54 (22.75)	0.75 ^†^
	After	67.87 (27.07)	69.86 (29.71)	0.72 ^‡^
	*p*-value ^§^	0.94	0.53	

Data are expressed as mean (standard deviation). ^†^ Independent *t*-test. ^‡^ Analysis of covariance (ANCOVA) adjusted for baseline values. ^§^ Paired *t*-test.

**Table 3:** Biochemical parameters and liver steatosis grades of the study patients at baseline and after the 12-wk intervention.

Variables		Control (*n* = 30)	Calorie-Restricted (CR) (*n* = 30)	*p*-Value
ALT (IU/L)	Before	33.71 (20.36)	43.58 (26.38)	0.30 ^†^
	After	40.94 (28.81)	39.25 (24.21)	0.01 ^‡^
	MD (95%CI), *p*-value ^§^	7.23 (−0.82 to 15.28), 0.07	−4.33 (−8.43 to −0.23), 0.04	
AST (IU/L)	Before	29.85 (9.80)	33.66 (12.62)	0.45 ^†^
	After	34.27 (21.06)	29.58 (12.57)	0.02 ^‡^
	MD (95%CI), *p*-value ^§^	4.42 (−1.95 to 10.79), 0.16	−4.08 (−8.04 to −0.12), 0.04	
MDA (nmol/mL)	Before	1.70 (0.41)	1.77 (0.39)	0.82 ^†^
	After	1.67 (0.54)	1.70 (0.42)	0.85 ^‡^
	MD (95%CI), *p*-value ^§^	−0.03 (−0.30, 0.24), 0.80	−0.07 (−0.29, 0.15), 0.51	
TAC (mmol/L)	Before	1.70 (0.49)	2.06 (0.36)	0.008 ^†^
	After	1.90 (0.42)	1.97 (0.34)	0.22 ^‡^
	MD (95%CI), *p*-value ^§^	0.20 (−0.01, 0.42), 0.07	−0.08 (−0.20, 0.04), 0.18	
SOD (U/g Hb)	Before	1179.23 (166.30)	1185.09 (152.08)	0.99 ^†^
	After	1164.32 (145.84)	1197.35 (167.57)	0.25 ^‡^
	MD (95%CI), *p*-value ^§^	−14.91 (−48.49, 18.66), 0.37	12.26 (−29.30, 53.81), 0.54	
GSH-Px (U/g Hb)	Before	50.07 (16.12)	50.50 (17.74)	0.99 ^†^
	After	49.83 (17.82)	48.04 (18.18)	0.35 ^‡^
	MD (95%CI), *p*-value ^§^	−0.24 (−2.42, 1.93), 0.82	−2.45 (−6.59, 1.68), 0.23	
FGF-21 (pg/mL) ^¥^	Before	392.2 (56.7, 2178.0)	549.4 (221.7, 2366.0)	0.18 ^†^
	After	381.9 (58.3, 1876.0)	531.7 (102.2, 2463.0)	0.95 ^‡^
	*p*-value^§^	0.54	0.87	
FBS (mg/dL)	Before	88.80 (8.45)	91.37 (11.34)	0.57 ^†^
	After	91.00 (7.15)	95.39 (10.90)	0.14 ^‡^
	MD (95%CI), *p*-value ^§^	2.20 (−1.34 to 5.74), 0.21	4.02 (0.48 to 7.54), 0.03	
TG (mg/dL)	Before	159.98 (86.91)	169.50 (111.81)	0.93 ^†^
	After	171.46 (97.38)	141.83 (67.37)	0.11 ^‡^
	MD (95%CI), *p*-value ^§^	11.47 (−21.01 to 43.96), 0.47	−27.66 (−59.22 to 13.89), 0.11	
TC (mg/dL)	Before	207.44 (36.62)	189.29 (40.80)	0.24 ^†^
	After	211.39 (34.48)	183.58 (36.47)	0.03 ^‡^
	MD (95%CI), *p*-value ^§^	3.94 (−6.46 to 14.35), 0.44	−5.70 (−15.53 to 4.11), 0.24	
LDL-C (mg/dL)	Before	135.75 (31.42)	118.22 (41.33)	0.21 ^†^
	After	139.41 (32.12)	118.21 (32.01)	0.15 ^‡^
	MD (95%CI), *p*-value ^§^	3.65 (−5.81 to 13.11), 0.43	−0.01 (−14.55 to 14.54), 0.99	
HDL-C (mg/dL)	Before	39.69 (6.78)	37.16 (10.71)	0.59 ^†^
	After	37.69 (8.55)	36.00 (10.48)	0.89 ^‡^
	MD (95%CI), *p*-value ^§^	−2.00 (−5.07 to 1.07), 0.19	−1.16 (−4.90 to 2.57), 0.52	
Grade of liver steatosis (0/1/2/3) ^€^	Before	0/12/18/0	0/10/17/3	0.21 *
	After	0/11/19/0	0/11/17/2	0.35 *

Data are expressed as mean (standard deviation) or geometric mean (min, max). ALT: alanine aminotransferase, AST: aspartate aminotransferase, MDA: malondialdehyde, TAC: total antioxidant capacity, SOD: superoxide dismutase, GSH-Px: glutathione peroxidase, FGF-21: fibroblast growth factor 21, FBS: fasting blood sugar, TG: triglycerides, TC: total cholesterol, LDL-C: low-density lipoprotein cholesterol, HDL-C: high-density lipoprotein cholesterol, MD (%95 CI): mean difference (%95 confidence interval). ^†^ Independent *t*-test. ^‡^ Analysis of covariance (ANCOVA) adjusted for baseline values and physical activity level changes. ^§^ Paired *t*-test. ^¥^ Analyzed after log transformation. ^€^ Number of patients in each grade according to ultrasound assay. * Fisher’s exact test.

## Data Availability

The datasets generated and analyzed during the current study are not publicly available but are available from the corresponding author on reasonable request.

## Supplementary Materials

The following supporting information can be downloaded at: https://www.mdpi.com/article/10.3390/nu14122509/s1, Table S1: CONSORT 2010 checklist of information to include when reporting a randomized trial *.
